# Does the biomarker search paradigm need re-booting?

**DOI:** 10.1186/1471-2490-9-1

**Published:** 2009-02-27

**Authors:** Robert E Hurst

**Affiliations:** 1 Departments of Urology, Biochemistry and Molecular Biology, and Environmental Health Sciences, Oklahoma University Health Sciences Center, 940 S.L. Young Blvd, Oklahoma City, OK 73104, USA

## Abstract

The clinical problem of bladder cancer is its high recurrence and progression, and that the most sensitive and specific means of monitoring is cystoscopy, which is invasive and has poor patient compliance. Biomarkers for recurrence and progression could make a great contribution, but in spite of decades of research, no biomarkers are commercially available with the requisite sensitivity and specificity. In the post-genomic age, the means to search the entire genome for biomarkers has become available, but the conventional approaches to biomarker discovery are entirely inadequate to yield results with the new technology. Finding clinically useful biomarker panels with sensitivity and specificity equal to that of cystoscopy is a problem of systems biology.

Bladder cancer is the most expensive cancer to manage from diagnosis to death from any cause [1], mainly because bladder cancer also is one of the most recurrent cancers, with some studies showing over half of patients will recur within 5 years [2]. The most widely held etiologic hypothesis is that superficial bladder cancer arises from mutations in fibroblastic growth factor 2 receptor and ras signaling, whereas the aggressive track has been thought to arise from mutations in the p53 and Rb signaling system [3]. Even though over 75% of bladder cancers are superficial at initial diagnosis, the problem of recurrence is particularly insidious because some 15 to 25% of patients progress to aggressive invasive disease [3] that may be responsible for half the deaths from bladder cancer. Poor patient compliance with cystoscopic monitoring begs for noninvasive biomarkers with sensitivity near 95%. Anything less than 95% sensitivity is asking patients to bet their lives on a test with worse sensitivity than the routine "gold standard" of cystoscopy. No product has reached the commercial market that meets these criteria [4]. Screening of high risk populations, such as smokers or workers exposed to industrial bladder carcinogens, requires high specificity to control costs but can be cost-effective even when sensitivity is 50% or less [5,6]. Prognostic markers to detect disease that is progressing need to emphasize sensitivity over specificity.

Because urine is readily available and contains both cells exfoliated from the normal and pathological urothelium as well as proteins from either secretion or cell lysis, bladder cancers have been early targets for biomarker development. Historically, tumor-associated antigens (TAA) and markers of abnormal ploidy using exfoliated cells were targeted. Cytology itself, although highly specific, lacks sensitivity [4]. Some of the TAA biomarkers such as M344, DD23 and 19A211 achieved excellent sensitivity when combined with abnormal ploidy markers such as chromosomal loss or rare event cells with > 5C DNA. Quantitative fluorescence image analysis techniques combining two or three markers have actually reached the requisite sensitivity and specificity [7]. Technology platforms have been barriers to commercialization because quantitation and rare event detection were both seen as keys to improving sensitivity and specificity, and until recently the technology for quantitative fluorescence microscopy has not been widely available.

Several products designed to detect proteins in urine have reached commercialization as dipstick tests. While these are cheap and convenient, the sensitivity tends to be insufficient, about 70% or less [8]. Although such tests might be useful as screening tests in high-risk populations, the need to follow up numerous false positives might render them less than cost effective. As tests for recurrence, a false sense of security from a negative could be fatal. Thus, the value of these tests is questionable.

In recent years, the development of microarrays and proteomics has brought the power of whole genome analysis to the field of biomarkers. Naively, many in the field have assumed that high-dimension studies of patient samples will magically yield robust, sensitive and specific biomarkers. Misconceptions about the technology and misunderstandings of the underlying biology of disease have plagued the field. Instead of there being "pathways" in which signaling is linear and definable, the reality is a large, interconnected network of cooperating proteins that regulate cellular growth, death and differentiation. Alterations in this network tend to ripple outward in unpredictable ways. Moreover, this complex system responds to complex inputs from the local tumor environment as well as all other biological variables affecting the organism. Individual molecular markers tend to lack sensitivity and specificity because, unlike morphologically-based grading, they are imperfectly reflective of the overall phenotype and overly sensitive to the cellular network. At a practical level, this means the probability of finding a single biomarker with the requisite sensitivity and specificity is vanishingly small.

Currently, high dimension studies are used to identify individual biomarkers that are then preliminarily validated in small studies [9], as has been done for decades. However, what is needed is not more studies of individual biomarkers but rather to determine how biomarkers relate to other biomarkers and how they can be combined into practical panels. Surprising to many, practical panels using large gene number "signatures" are unlikely to come from high-dimension studies of patient cancer tissues. This approach as applied to breast cancer was recently critiqued by Ein-Dor and colleagues, who showed that eight successive sets of 80 genes performed as well as did the original 80 chosen because they had the highest correlation with survival [10]. Moreover, such large biomarker sets tend not to be robust. Every patient's cancer is unique, and thousands of samples may be required to obtain robust biomarker panels [11]. Because the number of unknowns (genes) far exceeds the number of equations (patients) and the relationships are nonlinear, there are many solutions. The contributions of other biological variables cannot easily be dissected out, nor are the measurements (gene expression) independent of each other. Practical biomarker panels need to be constructed from small numbers of assays that are as independent as possible and reflective of the overall phenotype, rather than being a particular molecule that is altered in some fraction of cancers. In theory, developing a panel that assays all relevant branches of the complex cellular network might be possible, but this approach would be very difficult. Causality is manifested in complex ways in systems and the old assumptions of cause and effect are not adequate guides to use this new technology. These conclusions will require a virtual "re-boot" of the approach to biomarker development.

Perhaps the search for candidate biomarkers needs to be divorced from the validation in clinical populations. Analyzing patient samples with high-dimension approaches and expecting to find the single "magic bullet" biomarker, or even a small set of effective biomarkers, is unrealistic for the reasons discussed above. Only in model systems can reproducible samples be obtained and extraneous biology controlled. However, the models must be more realistic than cells cultured on plastic because many of the features reflecting the complex system of genes and proteins are inactive in cells grown on plastic [12]. Three-dimensional culture models involving an extracellular stromal element [12,13] should be more effective. The value of high-dimension studies of patient cancer specimens is less as discovery tools than as validation tools. When stored in public databases, they can be used *a priori *to test combinations of biomarkers derived independently from controlled, model system studies. A marker with 70% sensitivity could be useful if a second marker could be found that was positive in the marker 1-negative group who had disease. New mathematical models will be needed to classify biomarkers into independent or interacting sets using, for example, Bayesian logic trees (logistic regression) or nonlinear models adapted to large data sets and incorporating modern post-genomic bioinformatics and functional genomics that can determine associations between disease or phenotype and gene/protein expression [14,15].

After spending huge sums of money over the years on biomarker research there is remarkably little to show for the effort. The needs for clinically effective biomarkers for bladder cancer and other cancers are great, and only a fresh approach based on the powerful new technologies available recently are needed. However, along with new technology must come a new understanding that old paradigms are not adequate in the post-genomic age. To summarize, the new paradigm should consist of the following: further improvement in understanding of the complex, interacting system of genes and proteins to be able to develop relevant tests for dysregulation of the system, further development of mathematical techniques to analyze data within the system paradigm, and a high quality set of clinical studies using genome-wide techniques that captures the spectrum of disease. The intelligent development of biomarkers truly is a problem in systems biology.

## Pre-publication history

The pre-publication history for this paper can be accessed here:


